# Literature survey on epidemiology and pathology of gangliocytic paraganglioma

**DOI:** 10.1186/1471-2407-11-187

**Published:** 2011-05-20

**Authors:** Yoichiro Okubo, Megumi Wakayama, Tetsuo Nemoto, Kanako Kitahara, Haruo Nakayama, Kazutoshi Shibuya, Tomoyuki Yokose, Manabu Yamada, Kayoko Shimodaira, Daisuke Sasai, Takao Ishiwatari, Masaru Tsuchiya, Nobuyuki Hiruta

**Affiliations:** 1 Department of Surgical Pathology, Toho University School of Medicine, 6-11-1 Omori-Nishi, Ota-Ku, Tokyo, 143-8541, Japan; 2 Department of Neurosurgery, Toho University Ohashi Medical Center, 2-17-6, Ohashi, Meguro, Tokyo, 153-8515, Japan; 3 Department of Pathology, Kanagawa Cancer Center, 1-1-2, Nakao, Asahi-Ku, Yokohama-city, Kanagawa, 245-0815, Japan; 4 Division of General and Gastroenterological Surgery, Department of Surgery (Omori), Toho University School of Medicine, 6-11-1 Omori-Nishi, Ota-Ku, Tokyo, 143-8541, Japan

## Abstract

**Background:**

Although gangliocytic paraganglioma (GP) has generally been regarded as a neuroendocrine tumor, its origin remains unclear. We therefore aimed to investigate the details of this disease by carefully analyzing and extracting common features of the disease as presented in selected publications.

**Methods:**

We searched for English and Japanese cases of GP using the PubMed and IgakuChuoZasshi databases on August 2010. We then extracted and sampled raw data from the selected publications and performed appropriate statistical analyses. Additionally, we evaluated the expression of hormone receptors based on our previously reported case.

**Results:**

192 patients with GP were retrieved from the databases. Patient ages ranged from 15 y to 84 y (mean: 52.3 y). The gender ratio was 114:76 (male to female, 2 not reported). Maximum diameter of the tumors ranged from 5.5 mm to 100 mm (mean: 25.0 mm). The duodenum (90.1%, 173/192) was found to be the most common site of the disease. In 173 patients with duodenal GP, gastrointestinal bleeding (45.1%, 78/173) was found to be the most common symptom of the disease, followed by abdominal pain (42.8%, 74/173), and anemia (14.5%, 25/173). Rate of lymph node metastasis was 6.9% (12/173). Our statistical analysis indicated that significant differences were found for gender between GP within the submucosal layer and exceeding the submucosal layer. Furthermore, our immunohistochemical evaluation showed that both epithelioid and pancreatic islet cells showed positive reactivity for progesterone receptors.

**Conclusions:**

Our literature survey revealed that there were many more cases of GP exceeding the submucosal layer than were expected. Meanwhile, our statistical analyses and immunohistochemical evaluation supported the following two hypotheses. First, vertical growth of GP might be affected by progesterone exposure. Second, the origin of GP might be pancreatic islet cells. However, it is strongly suspected that our data have been affected by publication bias and to confirm these hypotheses, further investigation is required.

## Background

Gangliocytic paraganglioma (GP) was first reported as ganglioneuroma by Dahl et al. in 1957 [1] and Kepes et al. named this disease entity as GP in 1971 [2]. Although GP has generally been regarded as a neuroendocrine tumor, some authors have reported that GP is a hamartoma developing in misplaced embryonic pancreatic tissue [3-5]. Despite these investigations, the origin of GP remains unclear. However, a few cases of GP showing lymph node metastasis are well known [Table 1], and we previously reported such a case [6]. That case prompted us to survey publications of GP to investigate mechanisms for GP. In the present study, we searched literature databases and selected publications of GP in English and Japanese from 1957 to the early trimester of 2010 in order to investigate details of the disease.

**Table 1:** Duodenal gangliocytic paraganglioma with lymph node metastasis

Reference	Year	Age (years)	Sex	Chief Clinical presentation	Size (mm)	Operation	Follow up (months)
Buchler et al. [50].	1985	50	Male	Gastrointestinal bleeding	30	Surgical intervention	NED 20
Inai et al. [51].	1989	17	Male	Hematoemesis	20	PD	NED 32
Hashimoto et al. [52].	1992	47	Male	Incidental findings	65	PD	NED 14
Takabayashi et al. [53].	1993	63	Female	Abdominal pain	32	PPPD	NED 24
Dookhan et al. [24].	1993	41	Male	Abdominal pain	25	Additional surgical intervention	Recurrence 11 years after first surgical intervention
Sundararajan et al. [54].	2003	67	Female	Incidental findings	50	PD	NED 9
Bucher et al. [55].	2004	31	Female	Anemia, subclinical jaundice	30	PPPD	NED 44
Wong et al. [25].	2005	49	Female	Melena	14	PPPD	NED 12
Witkiewicz et al. [36].	2007	38	Female	Abdominal pain	15	PPPD	NR
Mann et al. [56].	2009	17	Female	Abdominal pain, vomiting, weight loss	NR	PPPD	NR
Okubo et al. [6]	2010	61	Male	Epigastralgia, tarry stool	30	PPPD	NED 6
Saito et al. [46].	2010	28	Male	Gastrointestinal bleeding, anemia	17	PD	NED 12

NR: not reported, PD: pancreatoduodenectomy, PPPD: pylorus-preserving pancreaticoduodenectomy

NED: no evidence of disease

There were twelve cases of gangliocytic paraganglioma with lymph node metastasis.

Few studies reported the epidemiology, pathology, and clinical characteristics of the disease. We then evaluated the details of the disease by carefully analyzing and extracting common features of the disease as presented in these selected publications.

## Methods

We searched English and Japanese cases of GP published from 1957 to the early trimester of 2010 using the PubMed http://www.ncbi.nlm.nih.gov/pubmed/ and IgakuChuoZasshi http://www.jamas.or.jp/ databases on August 2010 by conducting a search of "gangliocytic paraganglioma" with case report options as an additional tool. As a result, 4574 English and 27 Japanese publications were retrieved.

We checked the abstracts and 73 English and 24 Japanese (total 97) publications were regarded as cases of GP and 4504 publications were excluded.

Since this disease entity has been reported under other names, prior to Kepes et al. naming it as a GP [2], such as "ganglioneuroma", "non-chromaffin paraganglioma", and "paraganglioneuroma", we checked the references of 97 selected publications.

Finally, we added 8 publications as GP reports because they met the following criteria:

1. The characteristic three components could be confirmed in the manuscript or a figure.

2. The paper was cited in other publications as a GP report.

These 105 publications contained reports on 192 patients with GP. In the present study, we conducted a non-systematic literature review using 173 patients with duodenal GP.

We extracted and sampled raw data from the selected publications, such as clinical findings (age, sex, clinical symptoms, operation method, and outcome), histopathological findings (site, maximum diameter of the tumor, diagnostic rate from biopsy before operation, the depth of the tumor invasion, and with or without lymph node metastasis), and immunohistochemical findings.

In addition, we performed appropriate statistical analyses using the extracted data. Statistical analyses were performed using the non-parametric Mann-Whitney U test or Chi-square test.

Differences were considered significant at P < 0.05.

Additionally, we evaluated gender differences based on the results of our statistical analyses. Moreover, we evaluated the expression of hormone receptors in relation to estrogen and progesterone based on our previously reported case [6].

Finally, we declare that our study may be affected by publication bias because it comprises a cumulating case series.

## Result

### Overall findings

192 patients with GP were retrieved from the PubMed and IgakuChuoZasshi databases. Patient ages ranged from 15 y to 84 y (mean: 52.3 y). The gender ratio was 114:76 (male to female, 2 not reported). Maximum diameter of the tumors ranged from 5.5 mm to 100 mm (mean: 25.0 mm). The duodenum (90.1%, 173/192) was found to be the most common site of the disease, followed by the low-level spinal cord (2.1%, 4/192) [7-10], respiratory system (2.1%, 4/192) [11-14], jejunum (1.6%, 3/192) [5,15,16], and esophagus (1.0%, 2/192) [17,18]. There were individual cases involving the stomach [5], appendix [19], retromediastinum [20], pancreas [21], and mature teratoma [22] (constituent of the lesion), as well as a case of double focus in the duodenum and pancreas [23] (0.5%, 1/192, respectively). In accordance with these findings, the present study focuses on the duodenal lesion, which represents the site for the overwhelming majority of cases of the disease.

### Clinical findings

173 patients with duodenal GP were retrieved from the PubMed and IgakuChuoZasshi databases. Patient ages ranged from 15 y to 84 y (mean: 52.6 y). The gender ratio was 102:69 (2 not reported).

Gastrointestinal bleeding (45.1%, 78/173) was found to be the most common symptom of the disease among the symptoms reported in the papers, followed by abdominal pain (42.8%, 74/173), and anemia (14.5%, 25/173). In contrast, biliary obstruction was extremely rare (4.6%, 8/173). The follow-up period ranged from 12 months to 96 months, and no death from GP was reported. With the exception of a patient that had part of a previous tumor at the initial operation [24], no recurrence has been reported. There is only one report of a patient who underwent radiotherapy after surgical intervention [25]. 15 patients underwent an endoscopic procedure for removal [26-40], and one patient required additional surgical intervention due to the presence of a tumor residue following her first endoscopic intervention [36].

### Histopathological findings

Histopathological findings from forceps biopsies performed prior to surgical intervention were described in 35 patients. Among these cases, 4 patients were correctly diagnosed as GP, 24 patients did not show evidence of tumor cells (specimens did not contain tumor cells), and 7 patients were diagnosed or suspected of having a different neuroendocrine tumor (3 carcinoid tumors, 2 paragangliomas, 1 ganglioneuroma, and 1 case involving atypical cells).

The maximum diameter of the tumors ranged from 5.5 mm to 100 mm (mean: 24.2 mm). The depth of the tumor invasion was described in 108 patients, comprising 42 patients with submucosal invasion, 62 with muscularis propria invasion, and 4 with connective tissue beneath the muscularis propria. In summary, 42 patients had GP within the submucosal layer and 66 patients had GP exceeding the submucosal layer. Moreover, 12 of these patients showed lymph node metastasis that involved one submucosal lesion [Table 1].

### Immunohistochemical findings

Representative findings for each of the three characteristic components of tumor cells are as follows. In epithelioid cells, neuron specific enolase (NSE) showed the highest positive rate (93.9%, 77/82), followed by synaptophysin (90.0%, 36/40), pancreatic polypeptide (PP; 89.7%, 70/78), somatostatin (81.8%, 63/77), chromogranin-A (67.4%, 60/89), cytokeratins (48.3%, 28/58), and serotonin (22.0%, 13/59).

In spindle-shaped cells, S-100 protein showed the highest positive rate (94.2%, 98/104), followed by NSE (84.0%, 63/75), and synaptophysin (64.7%, 22/34).

In ganglion-like cells, synaptophysin showed the highest positive rate (94.3%, 33/35), followed by NSE (84.0%, 63/75), somatostatin (44.1%, 30/68), and PP (22.2%, 16/72). Immunohistochemical findings are summarized in Table 2.

**Table 2:** Immunohistochemical findings for each of the three characteristic components of the tumor.

	Epithelioid cells	Spindle-shaped cells	Ganglion-like cells
S-100	3.8% (4/104)	94.2% (98/104)	9.3% (9/97)
Synaptophysin	90.0% (36/40)	64.7% (22/34)	94.3% (33/35)
Chromogranin A	67.4% (60/89)	7.2% (6/83)	15.4% (12/78)
Neuron specific enolase	93.9% (77/82)	84.0% (63/75)	84.0% (63/75)
Cytokeratins	48.3% (28/58)	4.0% (2/50)	3.9% (2/51)
Vimentin	14.3% (1/7)	50.0% (2/4)	0.0% (0/6)
Neuron fiber	20.9% (14/67)	66.7% (44/66)	24.6% (16/65)
Pancreatic polypeptide	89.7% (70/78)	0.0% (0/72)	22.2% (16/72)
Somatostatin	81.8% (63/77)	8.7% (6/69)	44.1% (30/68)
Serotonin	22.0% (13/59)	0.0% (0/55)	16.7% (9/54)
Vasoactive intestinal polypeptide	12.1% (4/33)	12.9% (4/31)	9.7% (3/31)
Glucagon	6.4% (3/47)	0.0% (0/44)	2.4% (1/42)
Gastrin	5.9% (4/68)	0.0% (0/64)	0.0% (0/63)
Insulin	2.2% (1/46)	0.0% (0/41)	0.0% (0/42)

Positive or negative findings from immunohistochemistry in 173 extracted duodenal gangliocytic paraganglioma are summarized.

### Comparative analysis of clinicopathological findings

To determine significant factors associated with the progression of the tumor, the following comparative analyses were carried out.

When we employed evidence of metastasis as an indicator of progression, a significant difference was found only for the ages of patients among the clinicopathological findings concerning those with and without lymph node metastasis (Mann-Whitney U test: p = 0.01), and patients with metastasis were significantly younger than those without metastasis. In contrast, no significant differences were found in the maximum diameter of the tumors or gender when comparing patients with and without lymph node metastasis (Mann-Whitney U test: p = 0.10 or Chi-square test: p = 0.55, respectively).

These results are summarized in Table 3.

**Table 3:** Comparison of clinicopathological findings between patients with and without lymph node metastasis.

	Patients with lymph node metastasis	Patients without lymph node metastasis	Statistical analysis
Number of patients	12	161	
Age (years)	17 to 67 (mean: 43.5)	15 to 84 (mean: 53.4)	Significant difference was found (Mann-Whitney U test: p = 0.01)
Maximum diameter of the tumor (mm)	5.5 to 65 (mean: 29.8)	10 to 100 (mean: 23.5)	No significant difference was found (Mann-Whitney U test: p = 0.10)
Gender (male to female)	6:6	96:63 (2 not reported)	No significant difference was found (Chi-square test: p = 0.55)

A significant difference was found for the ages of patients with and without lymph node metastasis (Mann-Whitney U test: p = 0.01), and patients with lymph node metastasis were significantly younger than those without lymph node metastasis. In contrast, no significant differences were found for maximum diameter of the tumor or gender in patients with and without lymph node metastasis (Mann-Whitney U test: p = 0.10 or Chi-square test: p = 0.55, respectively).

Furthermore, comparisons of clinicopathological findings revealed significant differences for both gender and the rate of lymph node metastasis in patients when comparing GP within the submucosal layer and exceeding the submucosal layer (Chi-square test: p = 0.02, 0.03, respectively). Namely, patients having GP exceeding the submucosal layer were predominantly female and showed a higher rate of lymph node metastasis. These results are summarized in Table 4.

**Table 4:** Comparison of clinicopathological findings between GP within and exceeding the submucosal layer.

	Gangliocytic paraganglioma within the submucosal layer	Gangliocytic paraganglioma exceeding the submucosal layer	Statistical analysis
Number of patients	42	66	
Age (years)	28 to 84 (mean: 54.1)	15 to 73 (mean: 50.0)	No significant difference was found (Mann-Whitney U test: p = 0.23)
Maximum diameter of the tumor (mm)	5.5 to 65 (mean: 21.7)	10 to 100 (mean: 26.8)	No significant difference was found (Mann-Whitney U test: p = 0.18)
Gender (male to female)	29:13	30:36	Significant difference was found (Chi-square test: p = 0.02)
Rate of lymph node metastasis	2.4% (1/42)	16.7% (11/66)	Significant difference was found (Chi-square test: p = 0.03)

Significant differences were found for gender and the rate of lymph node metastasis between patients with GP within the submucosal layer and exceeding the submucosal layer (Chi-square test: p = 0.02, 0.03, respectively).

However, no significant differences were found for the maximum diameter of the tumor or the age of a patient between males and females. In addition, Spearman's rank correlation coefficient was calculated to assess any potential relationship between the maximum diameter of the tumors and ages of patients, but no significant relationships were found.

### Immunohistochemical evaluation of female sex hormone receptors

**Figure 1:**
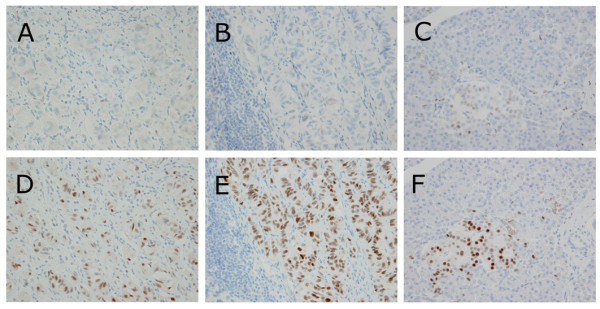
**Photomicrographs showing immunohistochemistry for estrogen and progesterone receptors**. (A and B) Epithelioid cells in both primary and metastatic foci were negative for estrogen receptors (× 400). (C) Pancreatic islet cells showed a weakly positive reactivity for estrogen receptos (× 400). (D and E) Epithelioid cells in both primary and metastatic foci showed a strongly positive reactivity for progesterone receptors (× 400). (F) Pancreatic islet cells showed a strongly positive reactivity for progesterone receptors (× 400).

Since the results mentioned above suggested that progression of the tumor has some relationship with sex hormone activities, we evaluated the expression of hormone receptors in relation to estrogen and progesterone based on our previously reported case of GP. As a result, epithelioid cells in both primary and metastatic foci showed strongly positive reactivity for progesterone receptors and negative reactivity for estrogen receptors [Figure 1]. In addition, normal pancreatic islet cells showed strongly positive reactivity for progesterone receptors and weakly positive reactivity for estrogen receptors [Figure 1].

## Discussion

We collected as many publications written in English or Japanese involving GP and examined them to extract characteristic features of the tumor as presented in clinical and histopathological findings.

We focused on determining the significant factors associated with the progression of the tumor. Here we discuss the clinical, histopathological, and immunohistochemical findings in reference to matters that emerged from our research.

In the present study, we found gastrointestinal bleeding as the most common symptom, followed by abdominal pain. This fact has been previously accepted and our data confirm it. All patients without autopsies and having clinically incidental focus had surgical interventions, including 15 patients with endoscopic intervention. Indeed, one patient required additional surgical intervention due to a residue of the tumor at her first endoscopic procedure [36], while other patients showed a good outcome without recurrence or metastasis. Furthermore, there was no record of a patient dying from GP, and patients with this tumor, therefore, have an extremely good prognosis. However, one patient has been reported as showing a recurrence due to a residue of a previous tumor at his initial surgical intervention [24]. Accordingly, we emphasize the importance of both a histopathological assessment of extensive tumor components at the surgical margin and imaging examinations to monitor for recurrence or metastasis after the operation.

Although one patient received irradiation after surgical intervention [25], we maintain that patients without residual tumor require no adjuvant therapy because no recurrence or metastasis has been reported in such patients. By contrast, it is still unclear whether a residual tumor can be controlled by irradiation or chemotherapy alone without surgical intervention.

The immunohistochemical findings on the tumors need consideration since the identification of three cellular components are essential for diagnosis. Epithelioid and ganglion-like cells showed a high positive rate for several kinds of immunohistochemical neuroendocrine markers, such as synaptophysin, chromogranin A, and NSE. In addition, epithelioid cells showed a high positive rate for PP. In contrast, spindle-shaped cells had the highest positive rate for S-100. These results are consistent with other previous publications. Furthermore, positive rates for each hormone, such as somatostatin, serotonin, gastrin, glucagon, and insulin in epithelioid cells, were significantly different and the meaning of this finding is worth investigating. However, these extracted immunohistochemical findings should be regarded as hints or suggestions because it is thought that results involving negative data have not been described in previous publications. In fact, our previous case report did not describe the immunohistochemical evaluation of each hormone according to negative reactivity.

Incidentally, we previously tried to establish the immunohistochemical prognostic indicators of GP using bcl-2, p53, and Ki-67, which are acceptable prognostic indicators in several kinds of neuroendocrine tumors [41-44]. However, all of these indicators showed a negative reactivity.

Unfortunately, there were no other cases of GP that included an immunohistochemical evaluation using bcl-2 and p53, and the value of these factors as prognostic indicators of GP remains unclear.

In contrast, two cases without lymph node metastasis were described with the Ki-67 labeling index in GP [45,46]. However, both showed extremely low Ki-67 labeling index values. Therefore, we suggest that immunohistochemical evaluation using Ki-67 may have a limited prognostic value in GP.

Finally, we gained insight into the progression of GP tumors and related factors. It has been accepted that GP usually arises from the submucosal or muscular layer, which may make the diagnosis difficult using a forceps biopsy prior to surgical intervention. In fact, we revealed that the diagnostic rate by biopsy before surgical intervention was only 11.4% (4/35). In addition, we showed that many more cases of GP exceeding the submucosal layer were reported (61.1%, 66/108) than expected, and GP exceeding the submucosal layer is a risk factor for lymph node metastasis. These facts emphasize the importance of imaging examinations prior to surgical interventions.

It is interesting to note that significant differences were found for gender between GP within the submucosal layer and exceeding the submucosal layer. On the basis of our investigation, a hypothesis emerged that asserted no significant relationship for gender and that female gender induces vertical growth of the tumor. To confirm part of our hypothesis, we focused on female-specific factors and initially evaluated tumor cells immunohistochemically using anti-estrogen and progesterone receptor antibodies. As a result, our immunohistochemical evaluation revealed that epithelioid cells showed positive reactivity for the progesterone receptor. Furthermore, some investigators reported that progesterone regulates neural differentiation [47,48]. These facts suggest that the vertical growth of GP might be affected by progesterone exposure.

Additionally, it is interesting to note that normal pancreatic islet cells also showed positive reactivity for the progesterone receptor. It has been reported that normal pancreatic islet cells and pancreatic neuroendocrine tumors showed positive reactivity for the progesterone receptor [49], and our literature survey demonstrated that epithelioid cells showed a high positive rate for PP (89.7%, 70/78) immunohistochemically. These facts indicate the relationship between GP and pancreatic islet cells. However, immunohistochemical evaluation for estrogen receptors differs between epithelioid cells and normal pancreatic islet cells, and our immunohistochemical evaluation was based on only one patient. Furthermore, it is strongly suspected that our data have been affected by publication bias.

To confirm these hypotheses, further investigation is required (e.g. compare the positivity between the metastatic cases [6,24,25,36,46,50-56] and non-metastatic cases for the immunohistochemical antigen expression).

## Conclusion

Our literature survey revealed that there were many more cases of GP exceeding the submucosal layer than were expected, and significant differences were found in gender between GP within the submucosal layer and exceeding the submucosal layer. Furthermore, our immunohistochemical evaluation showed that both epithelioid cells and normal pancreatic islet cells showed strongly positive reactivity for progesterone receptors. These immunohistochemical results support the following two hypotheses. First, vertical growth of GP might be affected by progesterone exposure. Second, the origin of GP might be the pancreatic islet cells. However, further investigation is required to confirm these hypotheses.

## Abbreviation

GP: gangliocytic paraganglioma; NSE: neuron specific enolase; PP: pancreatic polypeptide.

## Consent

Since the data of this study were extracted and sampled from previous publications, written informed consent for publication from patients does not exist with the exception of our previous publication. As for our previously reported cases, written informed consent was obtained from the patients for publication of this study as well as any accompanying images. A copy of the written consent is available for review by the Editor-in-Chief of this journal. Furthermore, the anonymity of all patients was strictly protected.

## Competing interests

Dr. Shibuya reports receiving research grants from Pfizer Inc., Janssen Pharmaceutical K.K., and Dainippon Sumitomo Pharma Co. All authors declare that they have no competing interests.

## Authors' contributions

YO conceptualized this study, integrated the data, and wrote the manuscript as a major contributor; MY carried out the histopathological evaluation and revised the manuscript; TN carried out statistical evaluation and revised the manuscript; KK and HN carried out statistical evaluation; KS gave final approval to the manuscript as a corresponding author; TY, MY, KS, DS, and TI sampled publications and extracted raw data from English and Japanese publications and integrated the data; MT advised the first author on gangliocytic paraganglioma as a clinical doctor; NH carried out histopathological and statistical evaluation and revised the manuscript as a last author. All authors contributed to conceptualizing and writing this study. Furthermore, all authors read and approved the final manuscript.

## Pre-publication history

The pre-publication history for this paper can be accessed here:

http://www.biomedcentral.com/1471-2407/11/187/prepub
